# Goat seropositivity as an indicator of Rift Valley fever (RVF) infection in human populations: A case-control study of the 2018 Rift Valley fever outbreak in Wajir County, Kenya

**DOI:** 10.1016/j.onehlt.2024.100921

**Published:** 2024-10-21

**Authors:** Ruth Omani, Lisa Cavalerie, Abukar Daud, Elizabeth A.J. Cook, Erenius Nakadio, Eric M. Fèvre, George Gitao, Jude Robinson, Mark Nanyingi, Matthew Baylis, Peter Kimeli, Joshua Onono

**Affiliations:** a International Livestock Research Institute, Nairobi, P.O. Box 30709-00100, Kenya; b Faculty of Veterinary Medicine, University of Liège, B-4000 Liège, Belgium; c University of Liverpool, Institute of Infection, Veterinary and Ecological Sciences, Liverpool L69 3BX, UK; d International Livestock Research Institute, Addis Ababa, P.O. Box 5689, Ethiopia; e County Department of Agriculture, Livestock & Fisheries, Wajir County Government, Kenya; f Directorate of Veterinary Services, Turkana County Government, Kenya; g Department of Veterinary Pathology, Microbiology and Parasitology, University of Nairobi, P.O. Box, 29053, Kangemi, Kenya; h University of Glasgow School of Social and Political Sciences, 42 Bute Gardens, University of Glasgow, Glasgow G12 8RT, UK; i Department of Clinical Studies, University of Nairobi, P.O. Box, 29053, Kangemi, Kenya; j Department of Public Health Pharmacology & Toxicology, University of Nairobi, P.O. Box, 29053, Kangemi, Kenya

**Keywords:** Rift Valley fever (RVF), Kenya, Serology, Prevalence, IgG, IgM, Human, Goat, Cattle, Camel, Case-control study

## Abstract

Rift Valley fever (RVF) is a viral zoonosis, which is considered as a threat to food security in the Horn of Africa. In Kenya, RVF is the 5th ranked priority zoonotic disease due to its high morbidity and mortality, frequent outbreak events, and associated socioeconomic impacts during outbreak events. In 2018, an RVF outbreak was confirmed in Kenya's Siaya, Wajir, and Marsabit counties. During this outbreak, 30 people were confirmed infected with RVF through laboratory tests; 21 in Wajir, 8 in Marsabit, and 1 in Siaya Counties.

Seventy-five (75) households (15 cases and 60 controls) were selected and interviewed using a case-control study design in 2021 (?). A case was a household with a member who was diagnosed with RVF in 2018. In addition, a total of 1029 animals were purposively selected within these households and serologically tested for RVF. The study aimed to estimate the contribution of various risk factors to RVF human occurrence in Kenya with a special focus on Wajir County. Wajir County was chosen due to high number of confirmed human cases reported in the 2018 outbreak. A univariable regression model revealed that owner-reported RVF virus exposure in livestock significantly increased the odds of an RVF human case in the household by 32.7 times (95 % CI 4.0–267.4). The respondent being linked to a goat flock that was IgG-positive increased the odds of an RVF human case by 3.8 times (95 % CI 1.17–12.3). In the final multivariable analysis, the respondent being linked to their own animals affected by RVF increased odds of having an RVF human case in the household by 56.9 times (95 % CI 4.6–700.4), while the respondent being linked to a neighbor household member affected decreased odds of having a RVF human case by 0.1 times (95 % CI 0.08–0.75).

In summary, these results have revealed a potential link for the spread of RVF infection from animals to humans in pastoralist households, hence it is critical to carry out targeted, community education, One Health surveillance, prevention, and control measures against the disease. This will be critical to protecting humans against potential spillovers of infections during outbreak events in livestock.

## 1 Introduction

In the Horn of Africa, more than 40 % [about 70 million] of people suffer from chronic food insecurity [1] due to a myriad of causes, including diseases affecting livestock. Rift Valley fever [RVF], a viral zoonosis, has become one of the region's most important constraints to food security. Its occurrence has direct implications for human and livestock health as well as indirect effects on the economy of the region, which is highly dependent on livestock trade, notably with the Middle East [2]. Indeed, RVF outbreaks can lead to restrictions of animal and human movements as well as trade embargos of live animals and livestock products [2,3]. In Kenya, frequent outbreaks have been reported from 1998 to 2021, with the 2007 outbreak leading to over 340 confirmed human cases, 90 deaths, and economic losses of over US$32 million [4,5].

Livestock such as goats, sheep, camels, and cattle often become infected with RVF virus when bitten by infected mosquitoes with the disease leading to high abortion rates in pregnant animals, often described as “abortion waves”, and high case fatality rates in young animals [2,3]. The majority of RVF-infected humans experience asymptomatic infection or mild fever that is self-limiting; chronic infection may lead to meningoencephalitis, ocular damage, and fatal hemorrhagic fever in a small proportion of cases [6,7]. Several paths of transmission of RVF to humans have been postulated during epidemics, with varying contributions to the epidemiologic profile [2,6,7]. Human infections are thought to occur through direct contact with blood or tissues of infected animals or inhalation of the aerosolised infective materials from infected animals during animal handling [2]. Humans may also get infected with RVF virus through bites from mosquitoes and other hematophagous flies [2,8]. Consumption of raw and unprocessed milk has also been epidemiologically linked to RVF disease in humans in several epidemics [2,7,9,10].

In Kenya, RVF is the 5th highest ranked priority zoonotic disease. This is due to its high morbidity and mortality, the regularity of outbreak events and the socioeconomic impacts during outbreak events [4,11]. Outbreaks are linked to heavy rainfall and flooding which deliver perfect conditions for mosquito vector multiplication and, subsequently, disease emergence. In 2018, an RVF outbreak was confirmed in Siaya, Wajir and Marsabit counties of Kenya. During this outbreak, 30 people were confirmed positive for RVF through laboratory tests; 21 in Wajir, 8 in Marsabit and 1 in Siaya Counties with a case fatality of 7 % [4,12,13]. Wajir and Marsabit counties are classified as regions with a high risk of RVF virus transmission. The counties have large numbers of nomadic pastoral communities and there is a high frequency of RVF outbreaks in the region [3,5]. The case from Siaya County [in southwestern Kenya] was the first ever to be reported in this region which was previously classified as low risk of RVF transmission [5].

Using a case-control design, this study aimed to estimate the contribution of various risk factors to human RVF infection during the 2018 outbreak in Wajir, Kenya. Our results contribute to consolidated efforts for detection, prevention, preparedness, and response plans for RVF in Kenya and other pastoral areas subject to RVF circulation and re-emergence.

## 2 Materials and methods

### 2.1 Ethical considerations

The study was approved by the International Livestock Research Institute Institutional Research Ethics Committee [ILRI-IREC 2019–25], the National Commission for Science, Technology and Innovation [NACOSTI/P/21/8631] and the University of Liverpool institutional ethics committee board.

Human subjects provided written informed consent before being interviewed. The project was explained, and consent documents were read aloud in Kiswahili and interpreted to the local Somali language dialect in the presence of a witness, after which participants either signed or gave a thumbprint impression to confirm agreement to the interview request. Participation was entirely voluntary, and it was made clear that opting out was possible at any stage of the interview and after the interview was completed.

Livestock sampling protocols were approved by the ILRI Institutional Animal Care and Use Committee approval number ILRI-IACUC 2019–25]. ILRI IACUC is registered in Kenya and complies with the UK's Animals Scientific Procedures Act 1986. Consent was obtained from livestock owners before sampling.

### 2.2 Study area

Wajir county was purposively selected for this study due to the RVF outbreak that was confirmed in June 2018 [4,12,13]. During the 2018 RVF outbreak, human and livestock infections were reported in Wajir. There was a total of 21 laboratory-confirmed positive human cases, including six human fatalities. At the time of the study, the number of animals infected was not available. Wajir County is predominantly semi-arid with a hot desert climate, has a human population of >780,000 and an area of 55,841 km^2^. The county is predominantly rural and characterized by pastoralism with the main economic activity being livestock production. Wajir County has about 1.2 million camels, 0.9 million cattle, 2.1 million sheep, and 3.1 million goats [14].

### 2.3 Study design and Sample size

**Fig. 1:**
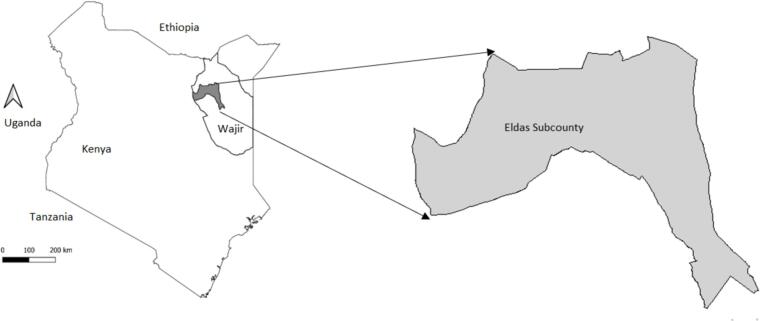
Map of Kenya showing the study sites at the border of Wajir county.

A case-control study design was used. A case was defined as a person, who was laboratory confirmed to have been infected with RVF by either the detection of anti-RVF IgM antibodies by IgM capture enzyme immunoassay or the detection of the RVF-specific genome by reverse transcription-polymerase chain reaction (RT-PCR) [15,16] in the 2018 RVF outbreak in Wajir county (See Fig. 1). As all cases were known to have kept livestock, and as our interest was in identifying what aspect of livestock ownership increased the risk of human RVF, we chose controls who were also livestock keepers. The control definition was, therefore, a person within the study area who did not show any clinical signs suspected or consistent with RVF during the same outbreak period but comes from a household with livestock and lived in the same locality as the case. The list of human confirmed RVF cases was obtained from the Wajir County Department of Public Health, Medical Services and Sanitation through the Wajir County Department of Agriculture, Livestock & Fisheries. As per the government hospital records and with guidance from staff from the county departments, active detection of the living human participants fitting the case definition were carried out. For each identified case, controls were selected from neighboring households (i.e, households within the same settlement area and within a 0.5 km radius) within the same village. In instances where the individual fitting the case definition was unavailable or dead, another adult household member was interviewed in their place.

Given the small and finite number of human cases available in Wajir [4], a 1:4 ratio of cases(15) to controls (60) was chosen to increase statistical power. Assuming a 50 % exposure rate of controls to risk factors, the study had approximately 33 % power to detect (with 95 % confidence) odds ratios of 2.6 and greater, 50 % power to detect odds ratios of 3.6 and greater and 80 % power to detect odds ratios of 7 and greater [https://sampsize.sourceforge.net/] [17,18]. Blood samples were collected from livestock owned by the case and control households to determine the presence of RVF seropositivity in each of the 75 selected herds. The livestock sample size was calculated using Epitools [19], based on an assumed finite population size of 200 animals per herd (cumulative herd size of sheep, goats, camels, and cattle), a design prevalence of 0.2, test sensitivity of 0.9, test specificity of 1, and a confidence level of 0.95. This gave a required sample size of 17 animals per herd which was rounded to 20 to allow for failures. The intention was to sample five animals per species (sheep, goats, camels, and cattle) across the 75 herds, making a total of 1500 animals.

### 2.4 Data and sample collection

The selected households were visited between June and August 2021. A questionnaire was administered to the participants to capture human and animal risk factors related to RVF exposure. The data collected included animal-level information (species and vaccination status) and respondent level information (age, occupation, and ownership of livestock). Other herd and household-level data collected included: the respondent's nature of contact with animals during the outbreak, the respondent's RVF status during the outbreak, and questions to assess their general knowledge of RVF disease.

In addition, 5 mL of whole blood was collected from the jugular vein of each selected animal for serum processing. Preference was given to animals that presented with clinical signs consistent with RVF disease during the 2018 outbreak and had not been vaccinated against RVF to ensure that the animals' seropositivity was not due to vaccination. Each evening, the blood tubes were centrifuged to separate serum that was later transferred to Eppendorf® vials. The serum was then frozen at −20 °C and transported on ice to the serology lab, Department of Public Health, Pharmacology and Toxicology, Faculty of Veterinary Medicine, University of Nairobi where it was stored at −20 °C until testing.

### 2.5 Laboratory analysis

The frozen sera were thawed to room temperature (18–26 °C). Competitive ELISA was performed using ID Screen® Rift Valley Fever Competition Multi-species kit (ID-vet, Grabels, France), which detects both IgG and IgM antibodies directed against the RVF virus nucleoprotein (NP). The competitive ELISA was done according to the manufacturer's instructions and as described previously [20,21]. The absorbance was read at 450 nm. The mean value of the two optical densities of the negative controls (OD_nc_) was computed, whereby a plate was considered valid if the OD_nc_ was >0.7. For a plate to be considered valid, the mean value of the two positive controls divided by OD_nc_ had to be <0.3. The competition percentage was calculated for each sample according to the eq. 100 x OD_sample_ / OD_nc_. If the value was ≤0.4, the sample was considered positive, while a value >0.5 was considered negative; values between 0.4 and 0.5 were inconclusive. Only samples that tested positive by competitive ELISA were tested for IgM antibodies using ID Screen® Rift Valley Fever IgM Capture (ID-vet, Grabels, France) according to the manufacturer's instructions and as described previously [22,23]. The presence of RVF virus-specific IgM was revealed eventually by the color reaction. Final absorbance was read at 450 nm, and the OD readings were converted to P*P* values (percentage of positive control serum) where PP values above 50 % were considered positive, values lower than or equal to 40 % were considered negative and values in between were considered inconclusive.

### 2.6 Data entry and statistical analysis

Questionnaire data and the laboratory results were entered into MS Excel (Microsoft Inc., Sacramento, California, USA) and then exported into Stata 15.1 (StataCorp LLC, College station, Texas, USA) for analyses. To begin with, the data were reviewed for accuracy after which they were coded, and descriptive statistics conducted. Proportions were evaluated for categorical variables and presented as a percentage of the overall number.

Univariable analysis using simple logistic regression was done to evaluate conditional associations with diagnosed RVF cases in humans from the 2018 RVF outbreak in Wajir County. The predictors were chosen from the available data based on a causal diagram and biological plausibility. Univariable associations with *p* ≤ 0.30 were eligible for multivariable analysis. Correlations between predictor variables were determined using pair-wise correlation. In cases where two or more variables were correlated (correlation coefficient > 0.5), statistical significance and biological plausibility were relied on to select the variable that would be included in the multivariable model.

Final models were developed by backward stepwise elimination of the variable with the highest *p*-value until all remaining variables had a p-value of <0.05. Explanatory variables were regarded as confounders if modification of the coefficients of the other significant variables by 30 % or more occurred when they were removed from the multivariable model. Likely biological interactions among significant explanatory variables in the final model were also evaluated, and the significant interactions were included in the final model [23]. The area under the curve of the receiver operating characteristic was used to evaluate the overall model performance.

## 3 Results

### 3.1 Household demographics and respondents, knowledge attitudes and practices related to RVF

A total of 75 households (15 cases and 60 controls) were interviewed from 1st to 25th of July 2021. The respondents were mainly herders and owners of animals, with a high proportion (*n* = 74, 98.7 %) having only informal education. Of the 15 cases, 9 had had confirmed exposure to RVF in 2018 and 8 shared a household with an individual who had had confirmed exposure to RVF in 2018 but was not available for interview. All households kept goats, with sheep being the second most owned animal species (*n* = 73, 97.3 %), followed by camels (*n* = 66, 88 %) and cattle being the least owned (*n* = 20, 26.7 %). The respondents were involved in managing their animals, with 68 (90.7 %) of the respondents confirming that they were involved in herding, 56 (74.7 %) in both slaughtering and birthing, and 51 (68 %) in milking. The characteristics of the respondents and the households were distributed between the cases and controls as seen in Table 1.

**Table 1:** Description of household characteristics and respondent's involvement in the management of livestock with relation to RVF human case during the 2018 outbreak in Wajir County, Kenya (univariable logistic mixed model among 75 households, 15 cases, 60 controls).

Variable	category	Number of respondent (proportion %)	Number of Respondents diagnosed RVF positive during the 2018 outbreak -, “cases” (Proportion of the category %)	Number of respondents not tested for RVF and who did not show disease symptoms - “controls” (Proportion of the category %)	OR (Odd Ratio)	95 % CI_OR inf_	95 % CI_OR sup_	P value
Respondent age	21–30	16 (21.3 %)	4 (26.7 %)	12 (20 %)	Baseline			
	31–40	19 (25,3 %)	6 (40 %)	13 (21.7 %)	1.38	0.31	6.14	0.67
	41–50	14 (18,7 %)	3 (20 %)	11 (18.3 %)	0.82	0.15	4.51	0.82
	Over 51	26 (34,7 %)	2 (13,3 %)	24 (40 %)	0.25	0.04	1.56	0.14*
Respondent education	Informal	74 (98.7 %)	14 (93.3 %)	60 (100 %)	Baseline			
	Postsecondary	1 (1.3 %)	1 (6.7 %)	0 (0 %)	N/A***	N/A	N/A	N/A***
Occupation of respondent	Herder and owner	68 (90.7 %)	12 (80 %)	56 (93,3 %)	Baseline			
	Herder	2 (2.7 %)	1 (1.7 %)	1 (1.7 %)	4.67	0.27	79.96	0.29*
	Owner	4 (5.3 %)	1 (1.7 %)	3 (5 %)	1.56	0.15	16.27	0.17*
	Trader	1 (1.3 %)	1 (1.7 %)	0 (0 %)	N/A***	N/A	N/A	N/A***
Household owns cattle	No	55 (73.3 %)	10 (66.7 %)	45 (75 %)	Baseline			
	Yes	20 (26.7 %)	5 (33.3 %)	15 (25 %)	1.5	0.44	5.09	0.52
Household owns sheep	No	2 (2.7 %)	0 (0 %)	2 (3.3 %)	Baseline****			****
	Yes	73 (97,3 %)	15 (100 %)	58 (96.7 %)	N/A	N/A	N/A	N/A
Household owns camel	No	9 (12 %)	2 (13.3 %)	7 (11.7 %)				
	Yes	66 (88 %)	13 (86.7 %)	53 (88.3 %)	0.89	0.16	4.63	0.86
Household own goat	No	0 (0 %)	0 (0 %)	0 (0 %)	Baseline			
	Yes	75 (100 %)	15 (100 %)	60 (100 %)	N/A	N/A	N/A	N/A
Respondent involved in milking	No	24 (32 %)	5 (33.3 %)	19 (31.7 %)	Baseline			
	Yes	51 (68 %)	10 (66.7)	41 (68.3 %)	0.93	0.29	3.09	0.90
Respondent involved in herding	No	7 (9.3 %)	2 (13.3 %)	5 (8.3 %)	Baseline			
	Yes	68 (90.7 %)	13 (86.7)	55 (91.7)	0.59	0.10	3.39	0.56
Respondent involved in birthing	No	19 (25.3 %)	4 (26.7 %)	15 (25 %)	Baseline			
	Yes	56 (74.7 %)	11 (73.3 %)	45 (75 %)	0.92	0.25	3.31	0.89
Respondent involved in slaughtering	No	19 (25.3 %)	6 (40 %)	13 (21.7 %)	Baseline			
	Yes	56 (74.7 %)	9 (60 %)	47 (78.3 %)	0.41*	0.12	1.38	0.15*
Respondent had contact with raw milk or raw meat during the outbreak	No	3 (4 %)	3 (20 %)	0 (0 %)	Baseline			
	Yes	72 (96 %)	12 (80 %)	60 (100 %)	N/A	N/A	N/A	N/A
Respondent consumed raw milk during the outbreak period	No	11 (14.7 %)	0 (0 %)	11 (18.3 %)	Baseline****			****
	Yes	64 (85.3 %)	15 (100 %)	49 (81.7 %)	N/A	N/A	N/A	N/A
Member of household affected by RVF	No one	59(78.7 %)	0(0 %)	59 (98.3)	Baseline			
	respondent	9(12 %)	9 (60 %)	0 (0 %)	N/A	N/A	N/A	N/A
	Alive relative	5 (6.7 %)	4 (26.7 %)	1 (1.7 %)	N/A	N/A	N/A	N/A
	Dead relative	2 (2.7 %)	2 (13.3 %)	0 (0 %)	N/A	N/A	N/A	N/A
Neighbor household member affected by RVF	No	62(82.7 %)	5(33.3 %)	57(95 %)	Baseline			
	Yes	13(17.3 %)	10(66.7 %)	3(5 %)	0.41*	0.13	1.28	0.12*
respondent experienced own animals affected by RVF (symptomatic)	No	43(57.3 %)	1(6.7 %)	42(70 %)	Baseline			
	Yes	32(42.7 %)	14(93.3 %)	18(30 %)	32.67**	3.99	267.44	0.01**
respondent neighbors' animals contracted RVF ((symptomatic)	No	22(29.3 %)	5(33.3 %)	17(28.3 %)	Baseline			
	Yes	53(70.7 %)	10(66.7 %)	43(71,7 %)	0.79	0.23	2.7	0.70

*p-value <0.3

** *p*-value<0.05

***This category perfectly predicts being an RVF case

****This category perfectly predicts not being an RVF case (means all respondents in that category were RVF cases, so odds ratios and confidence intervals can't be calculated due to no comparison group)

### 3.2 Livestock characteristics and seroprevalence

A total of 1029 animals were sampled from the 75 households described above. Species animal level seroprevalence was highest in camels and cattle at 15.2 % and 15.8 %, respectively and lowest in sheep and goats at 11.5 % and 10.6 %, respectively (Table 2). Only one animal (a sheep) was positive for IgM. Overall, herd seropositivity was highest in sheep at 38.7 % and lowest in goats at 34.7 % (Table 3).

**Table 2:** Sampled animals characteristics and seroprevalence.

Species	Nb of animals by Sex (Proportion %)		Age			IgG status Nb (Proportion %)		IgM seropositive (proportion %)
	F	M	Range	Mean	standard deviation	Positive	Negative	
Camel (*N* = 211)	126 (59.7 %)	85 (40.3 %)	[2,12]	8.0	1.8	32 (15.2 %)	179 (84.8 %)	0 (0 %)
Cattle (*N* = 76)	43 (56.6 %)	33 (43.4 %)	[3,10]	5.5	1.6	12 (15.8 %)	64 (84.2 %)	0 (0 %)
Goat (*N* = 376)	268 (71.3 %)	108 (28.7 %)	[1,11]	3.8	1.5	40 (10.6 %)	336 (89.4 %)	0 (0 %)
Sheep (*N* = 366)	272 (74.3 %)	94 (25.7 %)	[1,8]	4.2	1.4	42 (11.5 %)	324 (88.5 %)	1 (0.3 %)
Total (*N* = 1029)	709 (68.9 %)	320 (31.1 %)				126(12.2 %)	903 (87.8 %)	1 (0.1 %)

**Table 3:** Herd level characteristics by species and seroprevalence with relation to RVF human case during the 2018 outbreak in Wajir County, Kenya (univariable logistic mixed model among 75 households, 15 cases, 60 controls).

Species	Herd IgG status ⁎	frequency (%)	Number of Respondents diagnosed RVF positive during the 2018 outbreak, “cases” (Proportion of the category %)	Number of respondents not tested for RVF and who did not show disease symptoms (“controls”)	OR	95 % CI_OR inf_	95 % CI_OR sup_	*P* value
Camel (*n* = 75)	Negative	26(34.7 %)	6(40 %)	20(33.3 %)	Baseline			0.57
	Positive	18(24 %)	2(13.3 %)	16(26.7)	0.41	0.07	2.350435	0.32
	Not sampled⁎	31(41.3 %)	7(46.7 %)	24(40 %)	0.97	0.28	3.364443	0.97
Cattle (n = 75)	Negative	8 (53.3 %)	1(6.7 %)	7(11.7 %)	Baseline			0.75
	Positive	7(9.3 %)	2(13.3 %)	5(8.3 %)	2.8	0.19	40.06	0.62
	Not sampled⁎	60(80 %)	12(80 %)	48(80 %)	1.75	0.2	15.62	0.45
Goat (n = 75)	Negative	49(65.3 %)	6(40 %)	43(71.7 %)	Baseline			
	Positive	26(34.7 %)	9(60 %)	17(28.3 %)	3.79	1.17	12.29	**0.03**⁎⁎
Sheep (n = 75)	Negative	46(61.3 %)	8(53.3 %)	38(63.3 %)	Baseline			
	Positive	29(38.7 %)	7(46.7 %)	22(36.7 %)	1.51	0.482291	4.736183	0.48

Not sampled⁎ as there were no animals of that species in the heard.

⁎ A herd was considered positive if one animal was positive for IgG.

⁎⁎ P-VALUE < 0.05

### 3.3 Factors associated with RVF Positive human cases

At a significance level of *P* < 0.30, univariable logistic regression models (Tables 1) showed that being over 51 years old, being involved in slaughter and having a neighbor household member affected by RVF seemed to decrease odds of RVF seropositivity by 4 times (OR = 0.25, 95 % CI 0.04–1.56), 2.4 times (OR = 0.41, CI 95 % 0.12–1.38, *p* value = 0.15), and 2.4 times (OR = 0.41, CI 95 % 0.13–1.28, p value = 0.12) respectively. RVF virus exposure reported in livestock increased the odds of RVF in humans by 32.7 times (95 % CI 4.0–267.4, p value = 0.01). More specifically, IgG herd seropositivity in goats was associated with increased odds of RVF in human by 3.8 times (1.2,12.3, *p*-value = 0.03) (Table 3).

Some variables could not be used for univariable regression as all respondents from cases or controls fell in one single category making those perfect predictors of the case/control status: all the respondents who did not own sheep (*n* = 2), owning goats, and who consumed raw milk during the outbreak period (*n* = 11) were controls, making these categories perfect predictors of not being infected by RVF. On the contrary, all respondents with postsecondary education (n = 1), who were trader (n = 1) were cases, making these categories perfect predictors of being infected by RVF.

The factors associated with human RVF infection in the final multivariable analysis is shown in Table 4. The respondent's own animals affected by RVF increased odds of RVF seropositivity by 56.9 times (95 % CI 4.6–700.4). The respondent's neighbor being affected by RVF decreased odds of RVF seropositivity by by 10 times (OR = 0.1, 95 % CI 0.01–0.8).

**Table 4:** Final multivariable logistic mixed model for variables associated with human RVF seropositivity.

Variable	Category	Odds Ratio	Std. Err.	z	P > |z|	95 % Conf. Interval	
						LCL	UCL
respondent experienced own animals affected by RVF (symptomatic)	No	Baseline					
	Yes	56.9	72.88	3.15	0.002	4.6	700.4
Neighbor household member affected by RVF	No	Baseline					
	Yes	0.1	0.09	−2.71	0.027	0.01	0.8

## 4 Discussion

This case-control study reports risk factors associated with human infection with RVF disease in Wajir County of Kenya in cases from the 2018 RVF outbreak. Wajir County is an RVF hotspot in Kenya where humans, animals, and the environment interfaces are interlinked, highlighting the need for One Health approaches in RVF in research and routine surveillance.

A herd was considered positive if one animal turned positive for IgG; it has been documented that the presence of anti-RVF IgG antibodies in a single susceptible animal is often suggestive of RVF infection circulating in herds [24,25]. Overall herd seropositivity was high in sheep at 38.7 % (*n* = 29) and lowest in goats at 34.7 % (*n* = 26). Only one animal turned positive for IgM, a sheep. This finding also shows that at the time of sampling (July 2021) RVF virus was probably still in circulation, RVF had been detected in Kenya as from December 2020 and the virus was in circulation as per WHO's definition up to March 2021 [5,26]. It is possible that at the time of sampling the rest of the animals in the study areas either had undetected levels of IgM or had developed limited IgM responses towards the virus as has been described before [5,25]. It is also possible that the rest of the livestock had already transitioned to an “IgG state” as RVF IgM antibodies last up to 8 weeks post infection [[27], [28], [29]].These high herd level seropositivities of sheep and goat flocks suggests they may be good sentinels for RVF surveillance; a finding that has been documented in other studies [[30], [31], [32], [33]].

Univariate analysis revealed that several risk factors affect the likelihood of RVF seropositivity in humans. We found that RVF virus exposure reported in livestock significantly increased the odds of RVF virus seropositivity in humans. These findings are concurrent with what is known of RVF human infection; contact with infected animals during outbreaks is a risk factor to human infections [18,[34], [35], [36]]; RVF outbreaks in human beings tend to be clustered [8,[36], [37], [38]] and slaughtering infected animals exposes individuals to infected blood [8,18,[34], [35], [36]]. As pastoral livestock keepers have an in-depth knowledge of RVF symptoms in livestock [39,40]; this knowledge can be relied on for community-based syndromic diseases surveillance and to support early disease control interventions during possible RVF outbreaks [[40], [41], [42]]. In addition to the above risk factors, the respondent being linked to a goat flock that was IgG positive increased the odds of RVF seropositivity by 3.8 times (95 % CI 1.17–12.3). This finding agrees with a study that found that there was a weak positive correlation between goat and human seropositivity percentages [43]. Even though sheep are often considered the most susceptible species to RVF virus and regarded as the best sentinels for RVF surveillance [30,31,33], the finding that infection in goats is a risk factor for human infection implies that infections in goats could also be used to inform on the likelihood of disease in human populations.

In the multivariable analysis, the respondent having their own animals affected by RVF increased their odds of RVF seropositivity by more than fifty times. This finding indicates that direct contact with infected animals is a dominant factor to human RVF infection and hence that it is critical to carry out targeted one health surveillance, prevention, and control measures against the disease. Surprisingly, we also found that having a neighbor household member with RVF decreased the odds of the respondent having RVF by a factor of ten. This finding possibly denotes the multifaceted RVF virus transmission patterns, although no evidence exists indicating human to human transmission [46] and, in this case, a neighbor with RVF seems to be protective. While there are several possible explanations, it is possible that this result is an artefact of the study design, with controls being selected in the same village as cases; and hence uninfected respondents would often have had RVF-infected neighbors.

Our findings should be viewed considering several limitations. Based on the study's cross-sectional nature and on the potential life-long persistence of RVF IgG antibodies, it is not easy to establish the exact period of past exposure in animals. The presence of antibodies against RVF may be a result of antibody persistence in the host following initial exposure from a disease or from vaccination or may represent a cumulative effect from repeated exposure. We interpret the association of IgG seropositivity between animals and humans with caution because the exposure observed in the sampled humans may not necessarily have originated from the sampled animals. We also note that controls to the cases could not be completely randomized due to the lack of population/village registry. In addition to that, the interviews conducted relied on recall memory hence recall bias may have influenced responses regarding practices. However, the study still was able to generate valuable information of RVF exposure to humans in RVF-prone areas during RVF outbreaks.

## 5 Conclusion

In summary, this study found that at the time of sample collection, there was probable active outbreak detected in a sheep flock. We found that respondents' experiencing RVF exposure in livestock; slaughtering animals during outbreaks and owning goats were associated with increased odds of RVF seropositivity in humans. As per the final model, owning infected animals was a significant predictor to RVF seropositivity in humans.

Considering these findings, we recommend that community based syndromic diseases surveillance should be strengthened and relied upon during RVF outbreaks. Pastoralists have knowledge of various disease syndromes including RVF and may support country surveillance systems in early detection and mitigation. We also strongly suggest that the county should intensity continuous RVF disease surveillance to detect RVF outbreaks and, while doing so, there is need to rely on sheep and goats as sentinel species for surveillance as they have the highest levels of seropositivity. The Kenya livestock sector most importantly should invest in vaccinating livestock to mitigate against RVF. Finally, continuous stakeholder engagement should be done during RVF outbreak to sensitize communities and community leaders on infection prevention and control measures when handling animals and meat products.

## Author contributions

Conceptualization, R.O., E.F., E.C., G.G., J.O., J.R., L.C., M.N., and M.B.; methodology, R.O., E.F., E.N.,G.G., J.O., J.R., L.C., M.N., and M.B.; validation, R.O., and L.C.; formal analysis, J.O., R.O., and P·K; investigation, R.O., A.D., and E.R; resources, R.O., E.C., E.F., G.G., J.O., J.R., L.C., M.N., P.K., and M.B.; data curation, R.O., L.C., P.K., and E.N.; writing—original draft preparation, R.O., and P.K.; writing—review and editing, R.O., P.K., E.F., L.C., J.O., G.G and E.N.; supervision, R.O., E.C., E.F., G.G., J.O., J.R., L.C., M.N., and M.B.; project administration, R.O., E.N., and E.F; funding acquisition, E.F., J.O., J.R., and M.B. All authors have read and agreed to the published version of the manuscript.

The authors have not received any funding or benefits from industry or elsewhere to conduct this study.

## Funding

This work was funded by the Global Challenges Research Fund (GCRF) One Health Regional Network for the Horn of Africa (HORN) Project, from 10.13039/100014013UK Research and Innovation (UKRI) and 10.13039/501100000268Biotechnology and Biological Sciences Research Council (BBSRC) (project number BB/P027954/1).

## CRediT authorship contribution statement

**Ruth Omani:** Writing – review & editing, Writing – original draft, Visualization, Project administration, Methodology, Investigation, Funding acquisition, Formal analysis, Data curation, Conceptualization. **Lisa Cavalerie:** Writing – review & editing, Visualization, Supervision, Project administration, Methodology, Investigation, Formal analysis, Data curation, Conceptualization. **Abukar Daud:** Writing – review & editing, Methodology, Investigation. **Erenius Nakadio:** Writing – review & editing, Methodology, Investigation. **Eric M. Fèvre:** Writing – review & editing, Visualization, Validation, Supervision, Resources, Project administration, Investigation, Data curation, Conceptualization. **George Gitao:** Writing – review & editing, Validation, Supervision, Project administration, Methodology, Investigation, Data curation, Conceptualization. **Jude Robinson:** Writing – review & editing, Writing – original draft, Project administration, Methodology, Funding acquisition, Conceptualization. **Mark Nanyingi:** Writing – review & editing, Validation, Supervision, Investigation, Data curation, Conceptualization. **Matthew Baylis:** Writing – review & editing, Supervision, Resources, Project administration, Funding acquisition, Data curation, Conceptualization. **Peter Kimeli:** Writing – review & editing, Visualization, Validation, Methodology, Formal analysis, Data curation. **Joshua Onono:** Writing – review & editing, Supervision, Project administration, Methodology, Investigation, Funding acquisition, Conceptualization.

## Declaration of competing interest

I confirm that all authors of the manuscript have no conflict of interest to declare.

## Data Availability

Data will be made available on request.
